# Postoperative pulmonary complications of desflurane- versus sevoflurane-based general anesthesia in patients with chronic obstructive pulmonary disease or asthma undergoing gastrointestinal cancer surgery: a nationwide retrospective cohort study

**DOI:** 10.1007/s00540-025-03548-0

**Published:** 2025-07-16

**Authors:** Kanako Makito, Yuichiro Matsuo, Hiroki Matsui, Kiyohide Fushimi, Hideo Yasunaga

**Affiliations:** 1 Department of Biostatistics, School of Public Health, The University of Tokyo, 7-3-1, Hongo, Bunkyo-ku, Tokyo, 113-8655 Japan; 2 Department of Clinical Epidemiology and Health Economics, School of Public Health, The University of Tokyo, 7-3-1, Hongo, Bunkyo-ku, Tokyo, 113-0033 Japan; 3 Department of Health Policy and Informatics, Graduate School of Medical and Dental Sciences, Institute of Science Tokyo, S1560/S1568 M&D Tower 1-5-45 Yushima, Bunkyo-ku, Tokyo, 113-8519 Japan

**Keywords:** Chronic obstructive pulmonary disease, Asthma, Postoperative pulmonary complications, Volatile anesthetics

## Abstract

**Purpose:**

Desflurane and sevoflurane are widely used for general anesthesia; however, it remains uncertain if sevoflurane might be preferable for patients with chronic respiratory inflammatory diseases. This study compared postoperative outcomes of desflurane and sevoflurane following gastrointestinal cancer surgery in patients with chronic obstructive pulmonary disease (COPD) or asthma.

**Methods:**

We conducted a retrospective cohort study using the Japanese Diagnosis Procedure Combination database (April 2011–March 2022), identifying patients with COPD or asthma who underwent gastrointestinal cancer surgery. The primary outcome was postoperative pulmonary complications, including pneumonia, respiratory failure, mechanical ventilation > 24 h, and unplanned reintubation within 7 days after surgery. Secondary outcomes were in-hospital mortality and postoperative stay. We conducted propensity score overlap weighting and instrumental variable analyses adjusted for confounders.

**Results:**

We identified 24,243 COPD and 16,199 asthma patients. Propensity score overlap weighting showed no significant association between desflurane and increased postoperative pulmonary complications in COPD [adjusted risk difference (aRD) − 0.57%; 99% confidence interval (CI), − 1.8% to 0.60%] or asthma (aRD, − 0.62%; 99% CI, − 1.8% to 0.59%). In-hospital mortality did not differ significantly between groups in COPD (aRD, − 0.24%; 99% CI, − 0.76% to 0.29%) or asthma (aRD, 0.07%; 99% CI, − 0.45% to 0.59%). The postoperative stay also showed no significant association between the desflurane and sevoflurane groups.

**Conclusions:**

Desflurane-based anesthesia was not associated with increased postoperative pulmonary complications and mortality compared to sevoflurane in patients with chronic respiratory diseases undergoing gastrointestinal cancer surgery. However, further studies using reliable diagnostic criteria to assess COPD or asthma are warranted.

**Supplementary Information:**

The online version contains supplementary material available at 10.1007/s00540-025-03548-0.

## Introduction

Sevoflurane and desflurane are commonly used volatile anesthetics for maintaining anesthesia. While desflurane is generally recommended to avoid in patients with chronic respiratory inflammatory diseases, such as chronic obstructive pulmonary disease (COPD) and asthma, due to its high pungency [1–3], it is often clinically preferred owing to its faster recovery time following general anesthesia compared with sevoflurane [4, 5].

Volatile anesthetics have protective effects on myocardial and lung injury in experimental studies [6, 7]. Randomized controlled trials compared postoperative mortality and complications between total intravenous anesthesia (TIVA) and volatile anesthesia, mainly in patients who underwent thoracic surgery. A previous trial for on-pump coronary artery bypass grafting found that one-year mortality in the desflurane group (6.9%) was higher than that in the sevoflurane group (3.3%) but lower than that in the TIVA group (12.3%) [8]. Another trial for lung resection with one-lung ventilation showed no significant difference in postoperative pulmonary complications among patients under volatile anesthetics and propofol-based anesthesia [9].

In terms of the high airway irritation of desflurane, most studies compared the differences in adverse perioperative respiratory events, such as coughing and laryngospasm, between sevoflurane and desflurane in outpatient surgery. However, a few studies compared postoperative pulmonary complications among these volatile anesthetics in inpatient surgery. In a pooled analysis of a meta-analysis of randomized controlled trials that focused on respiratory adverse events, such as stridor, coughing, respiratory distress, and laryngospasm during general anesthesia in patients undergoing ambulatory surgery, desflurane is associated with more respiratory adverse events than sevoflurane [10]. On the other hand, a retrospective single-center cohort study in patients who underwent non-cardiac surgery demonstrated no significant difference in postoperative pulmonary complications between sevoflurane and desflurane, even in high-risk subgroups [11]. Anesthesia methods should be selected adequately to minimize perioperative complications in patients undergoing surgery. However, to our knowledge, there are no studies that compared postoperative pulmonary complications and mortality between sevoflurane and desflurane in patients with chronic respiratory inflammatory diseases.

This study aimed to compare postoperative pulmonary complications and in-hospital mortality between sevoflurane and desflurane in patients with chronic respiratory inflammatory diseases who underwent gastrointestinal cancer surgery. We hypothesized that desflurane is associated with a higher incidence of postoperative pulmonary complications than sevoflurane. Chronic respiratory inflammatory diseases mainly include obstructive pulmonary diseases, such as COPD and asthma; therefore, we assigned patients into two groups.

## Methods

The Institutional Review Board of the University of Tokyo approved this study (approval number: 3501-(5), approval date: October 20, 2011, renewal date: October 25, 2023), waiving the requirement for informed patient consent because of the data anonymity.

### Data source

We conducted a nationwide retrospective cohort study using inpatient data from the Japanese Diagnosis Procedure Combination database. Inpatient data have been collected annually since 2010 from > 1000 hospitals, accounting for over 50% of all inpatients who received acute care across Japan [12]. The database included information on age, sex, body height and weight, primary and comorbid diagnoses at admission and complications during hospitalization, Barthel Index [13] at admission and discharge, smoking status index, procedures (including procedures dates), duration of anesthesia, discharge status (discharge to home, discharge to other facilities, or in-hospital death), prescribed medications (including prescription dates, durations, and dose), admission and discharge dates, and hospital types (academic or non-academic). Primary comorbidity diagnoses and complications during hospitalization were coded with the International Statistical Classification of Diseases and Related Health Problems, 10th revision (ICD-10) codes, accompanied by text data in Japanese. The data related to patients' characteristics and diagnoses in the DPC database, which were required for determining inpatient medical fees, were entered by the attending physician according to the standardized manual issued by the government for DPC hospitals during in-hospital care. Medications prescribed and procedures performed during hospitalization were recorded when the physicians ordered them. This manuscript adheres to the STROBE guidelines.

### Patients selection

We constructed two cohorts (COPD or asthma) for all inpatients undergoing gastrointestinal cancer surgery between April 1, 2011, and March 31, 2022. We identified COPD and/or asthma from comorbidities at admission using ICD-10 codes (J41, J42, J43, J44, J440, J441 for COPD and J45, J46 for asthma). We excluded patients aged less than 18 years, those with both COPD and asthma, those who received TIVA, those who were intubated before surgery, and those who received a combination of sevoflurane and desflurane, and multiple surgeries during hospitalization.

### Exposure and outcomes

Desflurane and sevoflurane usage were the exposure and control variables, respectively. The primary outcome was postoperative pulmonary complications composed of bacterial pneumonia (ICD-10 codes: J12–18, J851), respiratory failure (J952, J953, J958, J959, J96), postoperative ventilation management for more than 24 h, and unplanned postoperative mechanical ventilation (newly started within 7 days following surgery). The secondary outcomes were in-hospital mortality and postoperative length of stay.

### Confounding variables

We extracted data on patient demographics at admission, surgical information, hospital characteristics, and intraoperative procedures as potential confounders. We defined a priori based on the literature review and the physiological plausibility of volatile agents and outcomes [11, 14–17]. We identified patient demographics at admission, including age, sex, body mass index, smoking index (daily number of cigarettes multiplied by years of smoking), Charlson comorbidity index [18], history of obstructive sleep apnea (G473), heart failure (I110, I130, I500, I501, I509), cancer stage, and Barthel index. Additionally, we collected data on preoperative oxygen therapy, preoperative renal replacement therapy, systemic steroid administration before and on the day of surgery, preoperative respiratory rehabilitation, emergent or non-emergent surgery, upper abdominal surgery or not, laparoscopic surgery or not, year of surgery, academic or non-academic hospital, hospital volume, duration of general anesthesia, intraoperative medications including vasopressor (dopamine, dobutamine, norepinephrine, epinephrine, and vasopressin), intermediate-acting neuromuscular blocking agents (rocuronium and vecuronium), opioids (morphine and fentanyl), reversal of neuromuscular blocking agents (sugammadex or neostigmine), epidural anesthesia, fluid therapy during surgery day, and blood transfusion during surgery day. We did not include dexamethasone as systemic steroid therapy on the day of surgery because dexamethasone is often used as postoperative nausea and vomiting prophylaxis.

Patients were assigned into four (18–39, 40–59, 60–79, and ≥ 80 years), four (< 18.5, 18.5–24.9, 25.0–29.9, and ≥ 30.0 kg/m^2^), three (0, 1–1000, and ≥ 1000), and three (100,75–95 and 0–70) categories based on age, body mass index, smoking index, and Barthel index, respectively. We defined hospital volume as the annual number of general surgeries performed under general anesthesia at each hospital and categorized the hospitals into two groups (high or low volume), classifying those with ≥ 4.5 and ≥ 3.0 annual surgeries under general anesthesia as high volume in the COPD or asthma cohorts, respectively. We classified cancer into four stages: 0 or I, II, III, and IV. We split types of gastrointestinal cancer into gastric, gallbladder, bile duct, hepatic, pancreatic, colon, and rectal cancers, and defined upper abdominal surgeries as those for these cancers except colon and rectal cancers. Surgical fiscal years were categorized into four groups (2011–2013, 2014–2016, 2017–2019, and 2020–2021).

### Statistical analysis

Propensity score overlap weighting was performed to account for the differences between the sevoflurane and desflurane groups. This method overcomes limitations related to the target population, balance, and precision associated with classical propensity score matching and inverse probability weighting by enabling the estimation of treatment effects in the population with the most treatment equipoise [19]. Overlap weighting is drawn by assigning weights based on the probability of not receiving (1 − propensity score) and receiving treatment (propensity score) for patients who receive the treatment and those who do not, respectively. We estimated propensity scores for receiving desflurane using a multivariable logistic regression model based on all the abovementioned variables.

The baseline demographics in each group were reported using numbers and proportions, and mean with standard deviation or median with interquartile range for categorical and continuous variables, respectively. We evaluated the baseline demographics balance between the two groups using standardized mean differences. Absolute standardized mean differences > 10% indicated a significant imbalance. Missing data on several variables, including body mass index, smoking index, cancer stage, and the Barthel index at admission, were treated with multiple imputations. We assumed that data were missing at random, created 20 multiple imputed datasets with chained equations, and combined all results and variances based on Rubin's rule [20, 21]. We also used complete case analyses as sensitivity analyses.

We calculated and compared risk differences for the outcomes of postoperative pulmonary complications and in-hospital mortality and absolute differences for postoperative length of stay in the overlap-weighted cohort after multiple imputations between the desflurane and sevoflurane groups. We employed a zero-truncated Poisson regression model to estimate the absolute differences in postoperative length of hospitalization. We used a clustered sandwich estimator to calculate standard errors, accounting for hospital-level clustering.

We conducted instrumental variable analyses, a substitute for treatment group randomization, to address the effect of unmeasured confounders because we did not observe intraoperative protective ventilation strategies, such as low tidal volumes, beneficial level of positive end-expiratory pressure, and alveolar recruitment maneuvers usage to prevent postoperative pulmonary complications and severity of chronic respiratory inflammatory diseases. Instrumental variables require the following conditions: (i) must be associated with the exposure, (ii) should not share common causes with the outcome, and (iii) should affect the outcome only through its effect on treatment but not directly influence the outcome [22, 23]. Volatile anesthetics usage fits these requirements, as it primarily depends on the physician’s preferences. Therefore, we used the desflurane usage proportion in each hospital as an instrumental variable. The proportion is the number of patients who received desflurane divided by the total number of patients in each hospital. We conducted two-stage residual inclusions and two-stage least squares to compare the primary and secondary outcomes between the sevoflurane and desflurane groups [24]. We tested the instrumental variable’s validity using a partial F-statistics test. We confirmed a partial *F* statistic > 10 (*P* < 0.001), suggesting that the instrumental variable was strongly associated with the treatment assignment [25]. We repeated instrumental variable analyses, adjusting for the same variables used in the propensity score estimation, using a dataset complemented by multiple imputations in the main analysis for comparison.

Additionally, we conducted subgroup analyses for COPD or asthma patients with severe conditions, which were defined as those who required oxygen therapy before surgery and/or systemic steroid therapy before and on the date of surgery. These analyses were performed using a propensity score overlap-weighted cohort based on the same variables as the primary analyses after multiple imputations were applied.

Data analyses were conducted using STATA software version 18.1 (StataCorp LP, TX, USA). We set the statistical significance level at *P* < 0.01 to control for type I errors due to multiple comparisons.

## Results

**Fig. 1:**
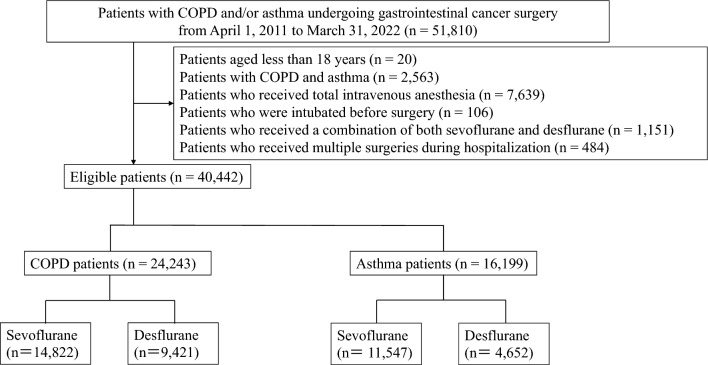
Flow diagram of patient selection. COPD, chronic obstructive pulmonary diseases; n, sample size

We identified patients with COPD and asthma who received gastrointestinal cancer surgery under general anesthesia during the study period. After excluding 11,368 patients, the eligible patients comprised 40,442 (24,243 patients in the COPD cohort and 16,199 patients in the asthma cohort). We divided each cohort into sevoflurane and desflurane groups (Fig. 1).

Table 1 illustrates the study population characteristics for each propensity score overlap-weighted cohort after multiple imputations. Overlap weighting balanced the distributions of all covariates between the two groups. In both groups, intermediate-acting neuromuscular blocking agents and opioids were used for general anesthesia maintenance in more than 90% of patients. Desflurane was more commonly used in academic and high-volume hospitals in both cohorts. Desflurane usage gradually increased over the study period (Table S1).

**Table 1:** Baseline characteristics of propensity score overlap-weighted cohorts after multiple imputations

	COPD cohort			Asthma cohort		
	Sevoflurane	Desflurane	ASD (%)	Sevoflurane	Desflurane	ASD (%)
Age, years, %						
18–39	0.12	0.12	0	0.72	0.72	0
40–59	4.7	4.7	0	10.5	10.5	0
60–79	67.2	67.2	0	60.9	60.9	0
≥ 80	28.0	28.0	0	27.8	27.8	0
Sex (male), %	85.2	85.2	0	53.2	53.2	0
Body mass index (kg/m^2^), %						
< 18.5	17.3	17.3	0	9.2	9.2	0
18.5–24.9	65.0	65.0	0	61.3	61.3	0
25.0–29.9	15.6	15.6	0	23.1	23.1	0
≥ 30.0	2.1	2.1	0	6.3	6.3	0
Smoking index, %						
0	28.5	28.5	0	59.6	59.6	0
1–1000	39.9	39.9	0	29.7	29.7	0
≥ 1000	31.6	31.6	0	10.6	10.6	0
Emergency admission, %	1.7	1.7	0	2.0	2.0	0
Charlson comorbidity index, median	3	3	0	3	3	0
Other comorbidities, %						
Obstructive sleep apnea	0.36	0.36	0	0.39	0.39	0
Chronic heart disease	5.0	5.0	0	4.1	4.1	0
Types of gastrointestinal cancer, %						
Gastric cancer	31.7	31.7	0	23.5	23.5	0
Gallbladder cancer	0.97	0.97	0	1.2	1.2	0
Bile duct cancer	0.95	0.95	0	1.2	1.2	0
Hepatic cancer	9.1	9.1	0	8.9	8.9	0
Pancreatic cancer	7.5	7.5	0	9.1	9.1	0
Colon cancer	31.4	31.4	0	38.2	38.2	0
Rectal cancer	18.4	18.4	0	17.9	17.9	0
Upper abdominal surgery, %	42.7	42.7	0	34.8	34.8	0
Laparoscopic surgery, %	49.3	49.3	0	53.0	53.0	0
Cancer stage, %						
0–I	30.8	30.8	0	31.1	31.1	0
II	32.2	32.2	0	31.1	31.1	0
III	27.5	27.5	0	27.8	27.8	0
IV	9.52	9.52	0	10.0	10.0	0
Barthel index, %						
100	87.8	87.8	0	87.2	87.2	0
75–95	5.2	5.2	0	5.7	5.7	0
0–70	7.0	7.0	0	7.1	7.1	0
Preoperative oxygen therapy, %	5.9	5.9	0	4.1	4.1	0
Preoperative hemodialysis, %	0.54	0.54	0	0.54	0.54	0
Academic hospital, %	18.1	18.1	0	17.9	17.9	0
Preoperative pulmonary rehabilitation, %	14.4	14.4	0	5.9	5.9	0
Use of systemic steroids before and during the surgery day, %	7.8	7.8	0	14.7	14.7	0
Anesthesia time, min, mean (SD)	348 (2.0)	348 (1.9)	0	346 (2.5)	346 (2.6)	0
Hospital volume, %						
Low	47.1	47.1	0	45.8	45.8	0
High	52.9	52.9	0	54.2	54.2	0
Fluid therapy during surgery day, ml, mean (SD)	7558 (50)	7558 (50)	0	7440 (67)	7440 (65)	0
Red blood cell transfusion during surgery day, units, %						
0	87.5	87.5	0	88.4	88.4	0
0–4	9.9	9.9	0	8.9	8.9	0
5–9	1.8	1.8	0	1.9	1.9	0
≥ 10	0.84	0.84	0	0.82	0.82	0
Use of vasopressor, %	20.1	20.1	0	17.5	17.5	0
Use of opioid, %	95.4	95.4	0	95.3	95.3	0
Use of epidural block, %	68.4	68.4	0	69.0	69.0	0
Use of intermediate-acting NMBA, %	99.8	99.8	0	100.0	100.0	0
Use of reverse intermediate-acting NMBA, %	92.2	92.2	0	92.6	92.6	0
Fiscal year, %						
2011–2013	10.2	10.2	0	9.4	9.4	0
2014–2016	34.7	34.7	0	30.7	30.7	0
2017–2019	35.2	35.2	0	36.5	36.5	0
2020–2021	19.8	19.8	0	23.4	23.4	0

COPD, chronic obstructive pulmonary disease; ASD, Absolute standardized difference; SD, standard deviation; NMBA, neuromuscular blocking agents

The proportions of postoperative pulmonary complications in patients with COPD were 7.5% (*n* = 1115) and 6.4% (*n* = 600) in the sevoflurane and desflurane groups, respectively. The proportions of postoperative pulmonary complications in patients with asthma were 5.5% (*n* = 630) and 4.5% (*n* = 209) in the sevoflurane and desflurane groups, respectively (Table 2). Table 3 presents the results of the primary and secondary outcomes in the overlap-weighted cohorts following multiple imputations. In patients with COPD, desflurane-based general anesthesia, when compared to sevoflurane-based anesthesia, was not associated with increased postoperative pulmonary complications [adjusted risk difference (aRD): − 0.57%, 99% confidence interval (CI) − 1.75% to 0.60%, *P* = 0.209] or in-hospital mortality (aRD: − 0.24%, 99% CI − 0.76% to 0.29%, *P* = 0.244). The asthma cohort showed no significant differences in postoperative pulmonary complications (aRD: − 0.62%, 99% CI − 1.82% to 0.59%, *P* = 0.187) or in-hospital mortality (aRD: 0.07%, 99% CI − 0.45% to 0.59%, *P* = 0.739) between the desflurane and sevoflurane groups. The instrumental variable analysis showed a significant association between desflurane usage and a decreased risk of postoperative pulmonary complications in patients with asthma (Table S2). Subgroup analyses limited to patients with severe COPD or asthma conditions found no significant association between desflurane usage and either postoperative pulmonary complications or in-hospital mortality (Table 4).

**Table 2:** Postoperative pulmonary complications and postoperative length of stay in patients with COPD or asthma

	COPD cohort (N = 24,243)				Asthma cohort (N = 16,199)			
	Sevoflurane (N = 14,822)		Desflurane (N = 9421)		Sevoflurane (N = 11,547)		Desflurane (N = 4652)	
Postoperative pulmonary complications, *n* (%)	1115	(7.5)	600	(6.4)	630	(5.5)	209	(4.5)
Bacterial pneumonia, * n* (%)	351	(2.4)	178	(1.9)	162	(1.4)	67.0	(1.4)
Respiratory failure, * n* (%)	590	(4.0)	342	(3.6)	354	(3.1)	119	(2.6)
Unplanned mechanical ventilation within 7 days after surgery, * n* (%)	106	(0.72)	53	(0.56)	44	(0.38)	15	(0.32)
Ventilation management for more than 24 h after surgery, * n* (%)	211	(1.4)	91	(0.97)	124	(1.1)	37	(0.80)
Postoperative length of stay (days), median (interquartile range)	16 (12–24)		15 (11–23)		15 (12–24)		15 (11–23)	

COPD, chronic obstructive pulmonary disease

**Table 3:** Association between desflurane and the outcomes in propensity score overlap-weighted cohorts after multiple imputations

	Desflurane (vs. sevoflurane)	99% confidence interval		*p* value
COPD				
Adjusted risk difference (%)				
Postoperative pulmonary complications	− 0.57	− 1.75	0.60	0.209
In-hospital mortality	− 0.24	− 0.76	0.29	0.244
Adjusted absolute difference (days)				
Postoperative length of stay	− 0.8	− 1.7	0.2	0.056
Asthma				
Adjusted risk difference (%)				
Postoperative pulmonary complications	− 0.62	− 1.82	0.59	0.187
In-hospital mortality	0.07	− 0.45	0.59	0.739
Adjusted absolute difference (days)				
Postoperative length of stay	0.3	− 0.9	1.4	0.568

COPD, chronic obstructive pulmonary disease

In terms of the postoperative length of stay, although the primary analyses revealed no significant association, the instrumental variable analyses in the COPD cohort showed that desflurane usage was significantly associated with a shorter postoperative length of stay compared with sevoflurane usage (Table S2). Similarly, the analysis for the patients with severe COPD conditions also showed that desflurane usage was more likely to decrease the postoperative length of stay (aRD: − 2.7 days, 99% CI − 5.3 days to − 0.2 days, *P* = 0.006) (Table 4). However, no significant associations were observed between desflurane and postoperative length of stay in patients with asthma, including those with severe conditions. In patients with severe COPD, the median postoperative length of stay was 17 (range 13–28 days) and 19 (range 13–34 days) days in the desflurane and sevoflurane groups, respectively (Table 5).

**Table 4:** Association between desflurane and the outcomes in propensity score overlap-weighted cohorts of patients with severe COPD or asthma after multiple imputations

	Desflurane (vs. sevoflurane)	99% confidence interval		*P* value
COPD				
Adjusted risk difference (%)				
Postoperative pulmonary complications	0.99	− 2.1	4.1	0.413
In-hospital mortality	− 1.2	− 3.2	0.83	0.132
Adjusted absolute difference (days)				
Postoperative length of stay	− 2.7	− 5.3	− 0.2	0.006*
Asthma				
Adjusted risk difference (%)				
Postoperative pulmonary complications	− 0.047	− 2.8	2.7	0.965
In-hospital mortality	0.25	− 1.5	2.0	0.719
Adjusted absolute difference (days)				
Postoperative length of stay	1.3	− 2.2	4.7	0.360

COPD, chronic obstructive pulmonary disease

*Significance at a *p* < 0.01 level

## Discussion

We assessed the association of volatile anesthetic agents with postoperative outcomes following gastrointestinal cancer surgery using a Japanese national database. Our results showed that desflurane, compared with sevoflurane, was not associated with an increased incidence of postoperative pulmonary complications or in-hospital mortality in patients with COPD or asthma, including those with severe disease, in the main analyses. The instrumental variable analyses revealed a significant association between desflurane and a shorter postoperative length of stay in patients with COPD, compared to sevoflurane. A similar trend was observed in analyses restricted to patients with severe COPD. In contrast, no significant differences in postoperative length of stay were observed between the desflurane and sevoflurane groups in the asthma cohort, nor in the main analysis of the COPD cohort.

**Table 5:** Postoperative pulmonary complications and postoperative length of stay in patients with severe COPD or asthma

	Severe COPD patients (N = 3265)				Severe asthma patients (N = 3294)			
	Sevoflurane (N = 2121)		Desflurane (N = 1144)		Sevoflurane (N = 2523)		Desflurane (N = 771)	
Postoperative pulmonary complications, * n* (%)	239	(11.3)	114	(10.0)	180	(7.1)	55	(7.1)
Bacterial pneumonia, * n* (%)	77	(3.6)	36	(3.2)	45	(1.8)	18	(2.3)
Respiratory failure, * n* (%)	100	(4.7)	58	(5.1)	87	(3.5)	27	(3.5)
Unplanned mechanical ventilation within 7 days after surgery, * n* (%)	26	(1.2)	6	(0.52)	16	(0.63)	5	(0.65)
Ventilation management for more than 24 h after surgery, * n* (%)	72	(3.4)	29	(2.5)	47	(1.9)	12	(1.6)
Postoperative length of stay (days), median (interquartile range)	19 (13–34)		17 (13–28)		17 (12–28)		17 (12–29)	

COPD, chronic obstructive pulmonary disease; N, sample size

A systematic review showed that the incidence of postoperative pulmonary complications following non-thoracic surgery ranged from 2.0 to 19.0%, although the target population and the definition of postoperative pulmonary complications varied among studies [26]. A study of 80 patients with COPD and stage IA lung cancer who underwent lung cancer surgery reported that 23 patients (28.8%) developed postoperative pulmonary complications [27]. The patients in our study were not limited to those with COPD and lung cancer who received lung resection, and the definition of postoperative pulmonary complications also differed from that in the previous study. The proportion of postoperative pulmonary complications in our study was lower than that in previous studies, owing to thoracic surgery’s contribution to postoperative pulmonary complications [28]. The proportion of postoperative pulmonary complications of patients with asthma in this study was comparable to that in a previous study, where it was 22 out of 257 patients (8.56%) [17].

Our findings revealed no significant association between desflurane and outcomes, including postoperative pulmonary complications, which aligns with the results of a previous study [11]. However, the previous study did not distinguish patients with chronic inflammatory respiratory disease from other high-risk patients. That study also did not focus on patients undergoing gastrointestinal cancer surgery, which is a common general surgical procedure usually accompanied by multiple risk factors for postoperative pulmonary complications. Therefore, our findings may provide important insights for selecting anesthetic agents in patients with these conditions.

The bronchodilator effects of isoflurane and sevoflurane are more pronounced in the peripheral airways than in the central airways [29, 30], and maintenance anesthesia with 1.1 minimum alveolar concentration (MAC) isoflurane or sevoflurane was shown to markedly reduce airway resistance in patients with COPD [31]. These effects can benefit patients with peripheral airway diseases such as COPD [30]. In a prospective observational study that evaluated the dose-dependent effects of volatile anesthetics, higher doses of volatile anesthetics were associated with lower odds of postoperative pulmonary complications and 30-day mortality compared with lower doses. These findings were consistent even when divided into desflurane, sevoflurane, and isoflurane groups [14]. Desflurane concentration associated with airway irritability during general anesthesia remains unclear. A study showed that desflurane concentrations at 1.0 MAC did not suppress upper airway reactivity [32], whereas another study demonstrated that desflurane concentrations ≥ 1.5 MAC were associated with airway irritation and increased airway resistance. [1, 2] In this study, we did not observe the volatile anesthetic concentration during general anesthesia. However, the reason for the lack of significant differences in primary outcomes between the two groups in this study may be that desflurane was not used at concentrations high enough to increase airway resistance under balanced general anesthesia.

Postoperative pulmonary complications are the most common postoperative complication and a major cause of perioperative mortality [33, 34] and are known to prolong the length of stay [34–36]. Although the main analysis showed no significant differences between the two groups, patients with COPD who received desflurane tended to have a shorter postoperative hospital stay compared to those who received sevoflurane in the instrumental variable analyses and the analysis restricted to patients with severe COPD. This may have been influenced by postoperative factors, which were not accounted for in the present analyses.

Inhalation anesthetic agents are potent greenhouse gases that contribute to the carbon footprint of operating services and the overall healthcare climate impact [37–39]. Notably, desflurane and nitrous oxide have much higher global warming potential than isoflurane or sevoflurane at clinically relevant concentrations [40]. In our analyses, desflurane usage in patients with COPD or asthma was not associated with worse postoperative outcomes. However, given its considerable environmental impact and lack of clinical advantage over sevoflurane, the use of desflurane in this population may not be justifiable.

The strength of this study is that we could estimate the association between volatile anesthesia agents and clinical outcomes, including postoperative pulmonary complications, in real-world clinical settings using large-scale data collected from acute care hospitals across Japan. It may be difficult to conduct randomized controlled trials due to the ethical issues regarding desflurane usage in patients with COPD or asthma. Moreover, we used multiple imputations for treating missing variables, which enabled all patients to be included in the analysis and provided valid estimates.

Our study has some limitations. First, the diagnoses of COPD, asthma, and postoperative pulmonary complications were based on the ICD-10 codes entered by the attending physician. The severity of these conditions was unclear because the DPC database does not encompass data on the results of laboratory tests, pulmonary function tests, arterial blood tests, and chest radiographs. Thus, there may have been misclassification bias. However, we found no association between desflurane usage and postoperative pulmonary complications or in-hospital mortality through a subgroup analysis limited to severe patients, and the results were consistent with those of the main analysis. Second, we addressed the biases derived from unmeasured confounders by using instrumental variable analyses. We used the proportion of desflurane usage by a hospital as a valid instrument variable based on the assumption that variations in desflurane usage across hospitals reflect differences in institutional or physician practices rather than patient characteristics. However, instrumental variable analyses can cause imprecise or biased estimates when the necessary conditions are not satisfied. When strong confounding exists, instrumental variables methods can be less useful because a strong instrument variable cannot be found, and assumptions are easily violated [41]. Moreover, in the present study, we used a nationwide database focusing only on Japan, and our findings may not be generalized to other healthcare systems or populations, particularly in countries with different healthcare infrastructures and recommendations about desflurane usage.

In conclusion, this large retrospective cohort study using a nationwide database in Japan found no significant association between desflurane-based general anesthesia and postoperative pulmonary complications or in-hospital mortality in patients with COPD or asthma. While desflurane usage did not show disadvantage in postoperative outcomes for patients with COPD or asthma, further studies that incorporate more reliable diagnostic criteria for COPD or asthma and assess pulmonary function severity through pulmonary function tests are needed.

## Supplementary Information

Below is the link to the electronic supplementary material.

Supplementary file1 (DOCX 41 KB)

Supplementary file2 (DOCX 24 KB)
