# Supplementary material for: Postoperative pulmonary complications of desflurane- versus sevoflurane-based general anesthesia in patients with chronic obstructive pulmonary disease or asthma undergoing gastrointestinal cancer surgery: a nationwide retrospective cohort study
Source: J Anesth. 2025 Jul 16;40(1):59–68. doi: 10.1007/s00540-025-03548-0 (PMC12860877; doi:10.1007/s00540-025-03548-0)
Supplement: Supplementary file 1 — Supplementary file1 (DOCX 41 KB) [file 540_2025_3548_MOESM1_ESM.docx]

Table S1. Baseline characteristics of patients with COPD and asthma

|  | COPD cohort (N=24,243) | | | | | Asthma cohort (N=16,199) | | | | |
| --- | --- | --- | --- | --- | --- | --- | --- | --- | --- | --- |
|  | Sevoflurane (N=14,822) | | Desflurane  (N=9,421) | | ASD  (%) | Sevoflurane,  (N=11,547) | | Desflurane  (N=4,652) | | ASD  (%) |
| Age, years, n(%) |  | |  | |  |  | |  | |  |
| 18–39 | 13 | (0.088) | 11 | (0.12) | 0.91 | 96 | (0.83) | 34 | (0.73) | 1.14 |
| 40–59 | 688 | (4.6) | 461 | (4.9) | 1.18 | 1314 | (11.4) | 467 | (10.0) | 4.34 |
| 60–79 | 10,075 | (68.0) | 6,277 | (66.6) | 2.87 | 7198 | (62.3) | 2808 | (60.4) | 4.06 |
| ≥80 | 4,046 | (27.3) | 2,672 | (28.4) | 2.38 | 2939 | (25.5) | 1343 | (28.9) | 7.69 |
| Sex (male), n(%) | 12,635 | (85.2) | 8,043 | (85.4) | 0.36 | 6,147 | (53.2) | 2,498 | (53.7) | 0.93 |
| Body mass index (kg/m^2^), n(%) |  |  |  |  |  |  |  |  |  |  |
| <18.5 | 2,700 | (18.2) | 1,581 | (16.8) | 3.78 | 1,167 | (10.1) | 414 | (8.9) | 4.12 |
| 18.5–24.9 | 9,544 | (64.4) | 6,081 | (64.6) | 0.38 | 6,909 | (59.8) | 2,832 | (60.9) | 2.13 |
| 25.0–29.9 | 2,185 | (14.7) | 1,475 | (15.7) | 2.55 | 2,723 | (23.6) | 1,056 | (22.7) | 2.09 |
| ≥30.0 | 256 | (1.73) | 223 | (2.37) | 4.52 | 621 | (5.4) | 311 | (6.7) | 5.49 |
| Missing | 137 | (0.92) | 61 | (0.65) | 3.14 | 127 | (1.1) | 39 | (0.84) | 2.67 |
| Smoking index, n(%) |  |  |  |  |  |  |  |  |  |  |
| 0 | 4,119 | (27.8) | 2,355 | (25.0) | 6.34 | 6,489 | (56.2) | 2,518 | (54.1) | 4.16 |
| 1–1000 | 5,143 | (34.7) | 3,493 | (37.1) | 4.96 | 3,047 | (26.4) | 1,292 | (27.8) | 3.12 |
| ≥1000 | 4,195 | (28.3) | 2,727 | (29.0) | 1.42 | 1,074 | (9.3) | 471 | (10.1) | 2.78 |
| Missing | 1,365 | (9.2) | 846 | (9.0) | 0.80 | 937 | (8.1) | 371 | (8.0) | 0.51 |
| Emergency admission, n(%) | 225 | (1.5) | 156 | (1.7) | 1.10 | 248 | (2.2) | 84 | (1.8) | 2.46 |
| Charlson comorbidity index, median (IQR) | 3(3-4) | | 3(3-4) | | 0 | 3(3-3) | | 3(3-3) | | 0 |
| Other comorbidities, n(%) |  |  |  |  |  |  |  |  |  |  |
| Obstructive sleep apnea | 44 | (0.30) | 40 | (0.43) | 2.13 | 30 | (0.26) | 22 | (0.47) | 3.53 |
| Chronic heart disease | 786 | (5.3) | 448 | (4.8) | 2.51 | 440 | (3.8) | 193 | (4.2) | 1.73 |
| Types of gastrointestinal cancer, n(%) |  |  |  |  |  |  |  |  |  |  |
| Gastric cancer | 4,903 | (33.1) | 2,935 | (31.2) | 4.12 | 2,821 | (24.4) | 1,083 | (23.3) | 2.70 |
| Gallbladder cancer | 145 | (0.98) | 94 | (1.0) | 0.20 | 145 | (1.2) | 54 | (1.2) | 0.87 |
| Bile duct cancer | 113 | (0.76) | 107 | (1.1) | 3.85 | 91 | (0.79) | 65 | (1.4) | 5.86 |
| Hepatic cancer | 1,126 | (7.6) | 965 | (10.2) | 9.29 | 837 | (7.3) | 459 | (9.9) | 9.37 |
| Pancreatic cancer | 898 | (6.1) | 813 | (8.6) | 9.87 | 851 | (7.4) | 460 | (9.9) | 8.98 |
| Colon cancer | 4,836 | (32.6) | 2818 | (29.9) | 5.86 | 4,642 | (40.2) | 1,725 | (37.1) | 6.41 |
| Rectal cancer | 2,801 | (18.9) | 1689 | (17.9) | 2.50 | 2,160 | (18.7) | 806 | (17.3) | 3.59 |
| Upper abdominal surgery, n(%) | 6,287 | (42.4) | 4,101 | (43.5) | 2.25 | 3,894 | (33.7) | 1,661 | (35.7) | 4.16 |
| Laparoscopic surgery, n(%) | 6,296 | (42.5) | 4,881 | (51.8) | 18.8 | 5,527 | (47.9) | 2,494 | (53.6) | 11.5 |
| Cancer stage, n(%) |  | |  | |  |  | |  | |  |
| 0–I | 3,460 | (23.3) | 2112 | (22.4) | 2.20 | 2,894 | (25.1) | 1,079 | (23.2) | 4.37 |
| II | 3,563 | (24.0) | 2,219 | (23.6) | 1.14 | 2,782 | (24.1) | 1,112 | (23.9) | 0.44 |
| III | 3,051 | (20.6) | 1,885 | (20.0) | 1.46 | 2,554 | (22.1) | 977 | (21.0) | 2.72 |
| IV | 1,176 | (7.9) | 606 | (6.4) | 5.82 | 932 | (8.1) | 344 | (7.4) | 2.53 |
| Missing | 3,570 | (24.1) | 2,599 | (27.6) | 8.01 | 2,385 | (20.7) | 1,140 | (24.5) | 9.22 |
| Barthel index, n(%) |  |  |  |  |  |  |  |  |  |  |
| 100 | 12,470 | (84.1) | 8,107 | (86.1) | 5.39 | 9,708 | (84.1) | 3,946 | (84.8) | 2.07 |
| 75–95 | 810 | (5.5) | 452 | (4.8) | 3.02 | 602 | (5.2) | 263 | (5.7) | 1.94 |
| 0–70 | 1068 | (7.2) | 591 | (6.3) | 3.72 | 864 | (7.5) | 311 | (6.7) | 3.11 |
| Missing | 474 | (3.2) | 271 | (2.9) | 1.87 | 373 | (3.2) | 132 | (2.8) | 2.29 |
| Preoperative oxygen therapy, n(%) | 998 | (6.7) | 506 | (5.4) | 5.72 | 522 | (4.5) | 187 | (4.0) | 2.48 |
| Preoperative hemodialysis, n(%) | 73 | (0.49) | 52 | (0.55) | 0.83 | 48 | (0.42) | 30 | (0.65) | 3.16 |
| Academic hospital, n(%) | 2124 | (14.3) | 2016 | (21.4) | 18.5 | 1,754 | (15.2) | 886 | (19.0) | 10.2 |
| Preoperative pulmonary rehabilitation, n(%) | 2411 | (16.3) | 1335 | (14.2) | 5.84 | 737 | (6.4) | 269 | (5.8) | 2.51 |
| Use of systemic steroids before and during the surgery day  , n(%) | 1267 | (8.6) | 686 | (7.3) | 4.69 | 2,163 | (18.7) | 629 | (13.5) | 7.87 |
| Anesthesia time, min, mean (SD) | 334(136) | | 358(137) | | 17.0 | 331(132) | | 351(145) | | 14.4 |
| Hospital volume, n(%) |  |  |  |  |  |  |  |  |  |  |
| Low | 7,989 | (53.9) | 3,990 | (42.4) | 23.3 | 5,869 | (50.8) | 2,023 | (43.5) | 14.7 |
| High | 6,833 | (46.1) | 5,431 | (57.6) | 23.3 | 5,678 | (49.2) | 2,629 | (56.5) | 14.7 |
| Fluid therapy during the surgery day, ml, mean (SD) | 7571(3524) | | 7,633(3581) | | 1.74 | 7,326(3464) | | 7,558(3652) | | 6.52 |
| Red blood cell transfusion during surgery day, units, n(%) |  |  |  |  |  |  |  |  |  |  |
| 0 | 12,841 | (86.6) | 8,288 | (88.0) | 4.02 | 10,218 | (88.5) | 4,096 | (88.0) | 1.37 |
| 0–4 | 1,546 | (10.4) | 907 | (9.6) | 2.67 | 1,034 | (9.0) | 426 | (9.2) | 0.71 |
| 5–9 | 311 | (2.1) | 153 | (1.62) | 3.51 | 219 | (1.9) | 86 | (1.9) | 0.35 |
| ≥10 | 124 | (0.84) | 73 | (0.76) | 0.69 | 76 | (0.66) | 44 | (0.95) | 3.23 |
| Use of vasopressor, n(%) | 3,041 | (20.5) | 1,966 | (20.9) | 0.87 | 2,001 | (17.3) | 846 | (18.2) | 2.24 |
| Use of opioid, n(%) | 13,960 | (94.2) | 9,026 | (95.8) | 7.45 | 10,773 | (93.3) | 4,459 | (95.9) | 11.3 |
| Use of epidural block, n(%) | 10,291 | (69.4) | 6,475 | (68.7) | 1.52 | 7,958 | (68.9) | 3,239 | (69.6) | 1.53 |
| Use of intermediate-acting NMBA, n(%) | 14,748 | (99.5) | 9,404 | (99.8) | 5.48 | 11,501 | (99.6) | 4,651 | (100) | 8.24 |
| Use of reverse intermediate-acting NMBA, n(%) | 13,221 | (89.2) | 8,808 | (93.5) | 15.3 | 10,225 | (88.6) | 4,351 | (93.5) | 17.5 |
| Fiscal year, n(%) |  |  |  |  |  |  |  |  |  |  |
| 2011–2013 | 4,592 | (31.0) | 590 | (6.3) | 67.0 | 3,741 | (32.4) | 304 | (6.5) | 69.1 |
| 2014–2016 | 4,609 | (31.1) | 2,972 | (31.6) | 0.97 | 3,291 | (28.5) | 1,300 | (27.9) | 1.24 |
| 2017–2019 | 3,586 | (24.2) | 3,803 | (40.4) | 35.1 | 2,821 | (24.4) | 1,839 | (39.5) | 32.8 |
| 2020–2021 | 2,035 | (13.7) | 2,056 | (21.8) | 21.3 | 1,694 | (14.7) | 1,209 | (26.0) | 28.4 |

COPD, chronic obstructive pulmonary disease; ASD, Absolute standardized difference; SD, standard deviation; IQR, interquartile range; NMBA, neuromuscular blocking agents.
