# Supplementary material for: Postoperative pulmonary complications of desflurane- versus sevoflurane-based general anesthesia in patients with chronic obstructive pulmonary disease or asthma undergoing gastrointestinal cancer surgery: a nationwide retrospective cohort study
Source: J Anesth. 2025 Jul 16;40(1):59–68. doi: 10.1007/s00540-025-03548-0 (PMC12860877; doi:10.1007/s00540-025-03548-0)
Supplement: Supplementary file 2 — Supplementary file2 (DOCX 24 KB) [file 540_2025_3548_MOESM2_ESM.docx]

Table S2. Associations between desflurane and the outcomes in sensitivity analyses

|  | Desflurane (vs. Sevoflurane) | 99% confidence interval | | p-value |
| --- | --- | --- | --- | --- |
| Complete case analyses |  |  |  |  |
| COPD |  |  |  |  |
| Adjusted risk difference (%) |  |  |  |  |
| Postoperative pulmonary complications | -0.75 | -2.0 | 0.54 | 0.134 |
| In-hospital mortality | -0.18 | -0.66 | 0.30 | 0.330 |
| Adjusted absolute difference (days) |  |  |  |  |
| Postoperative length of stay | -1.0 | -2.0 | 0.1 | 0.020 |
|  |  |  |  |  |
| Asthma |  |  |  |  |
| Adjusted risk difference (%) |  |  |  |  |
| Postoperative pulmonary complications | -0.54 | -1.8 | 0.68 | 0.254 |
| In-hospital mortality | -0.03 | -0.52 | 0.46 | 0.878 |
| Adjusted absolute difference (days) |  |  |  |  |
| Postoperative length of stay | 0.3 | -1.0 | 1.5 | 0.580 |
| Instrumental variable analyses before multiple imputations | | |  |  |
| COPD |  |  |  |  |
| Adjusted risk difference (%) |  |  |  |  |
| Postoperative pulmonary complications | -1.2 | -3.1 | 0.71 | 0.110 |
| In-hospital mortality | 0.29 | -0.70 | 1.28 | 0.447 |
| Adjusted absolute difference (days) |  |  |  |  |
| Postoperative length of stay | -2.1 | -3.8 | -0.3 | 0.002* |
|  |  |  |  |  |
| Asthma |  |  |  |  |
| Adjusted risk difference (%) |  |  |  |  |
| Postoperative pulmonary complications | -1.9 | -4.0 | 0.09 | 0.014 |
| In-hospital mortality | 0.16 | -1.0 | 1.3 | 0.721 |
| Adjusted absolute difference (days) |  |  |  |  |
| Postoperative length of stay | -0.5 | -2.3 | 1.3 | 0.485 |
| Instrumental variable analyses after multiple imputations | | |  |  |
| COPD |  |  |  |  |
| Adjusted risk difference (%) |  |  |  |  |
| Postoperative pulmonary complications | -0.83 | -2.4 | 0.73 | 0.186 |
| In-hospital mortality | -0.11 | -0.99 | 0.78 | 0.756 |
| Adjusted absolute difference (days) |  |  |  |  |
| Postoperative length of stay | -2.3 | -3.8 | -0.9 | 0.001* |
|  |  |  |  |  |
| Asthma |  |  |  |  |
| Adjusted risk difference (%) |  |  |  |  |
| Postoperative pulmonary complications | -2.1 | -3.9 | -0.34 | 0.006* |
| In-hospital mortality | 0.27 | -0.83 | 1.4 | 0.541 |
| Adjusted absolute difference (days) |  |  |  |  |
| Postoperative length of stay | 0.1 | -1.7 | 2.0 | 0.844 |

COPD, chronic obstructive pulmonary disease

* indicates significance at a p < 0.01 level.
